# What’s in a name? Context-dependent significance of ‘global’ methylation measures in human health and disease

**DOI:** 10.1186/s13148-017-0311-0

**Published:** 2017-01-13

**Authors:** Regan Vryer, Richard Saffery

**Affiliations:** 1 Murdoch Childrens Research Institute, 50 Flemington Rd, Parkville, Victoria 3052 Australia; 2 Department of Paediatrics, University of Melbourne, Parkville, Victoria Australia

## Abstract

The study of DNA methylation in development and disease has ‘exploded’ as a field in recent years, with three major classes of measurement now routine. These encompass (i) locus-specific, (ii) genome-scale/wide and (iii) ‘global’ methylation approaches. Measures of global methylation refer to the level of 5-methylcytosine (5mC) content in a sample relative to total cytosine. Despite this, several other measures are often referred to as ‘global’, with the underlying assumption that they accurately reflect 5mC content. The two most common surrogate, or proxy, measures include generating a mean or median methylation value from (i) the average measure in thousands of highly repetitive genomic elements and (ii) many thousands to several million primarily unique CpG sites throughout the genome. Numerous lines of evidence suggest the underlying assumption of equivalence of these measures is flawed, with considerable variation in the regulation of different ‘flavours’ of DNA methylation throughout the genome depending on cell type, differentiation and disease state. As such, the regulation of methylation ‘types’ is often uncoupled. The emerging picture suggests that no approach can accurately detect all biologically important differences in 5mC variation and distribution in all instances, with this needing to be ascertained on a case-by-case basis. Thus, it is important to clearly elaborate the genomic context and content of DNA methylation being analysed, the sample and developmental stage in which it is being examined and to remember that in most instances, the most common measures are not a true representation of ‘global’ 5mC content as orginally defined.

## Approaches

There are three general classes of DNA methylation measures. The first is locus/gene-specific analysis, usually at a small number of defined CpG sites in a limited genomic region. The second involves building a profile of DNA methylation by measuring many unique sites across the genome, (genome-wide or genome-scale analysis). The third, referred to as global methylation, is designed to assess the total 5-methylcytosine (5mC) content (but not 5hmC, 5fC or 5caC) within a sample using either direct or surrogate/proxy measures. It has been known for decades that changes in global methylation are a feature of human malignancy [1].

Global DNA methylation refers to the total level of 5mC content in a sample relative to total cytosine content. This is usually assessed using HPLC [1–3] but can also be assessed by HPLC coupled tandem mass spectrometry (LC-MS/MS) [4] and high-performance capillary electrophoresis [5]. These methods are the only true measures of global methylation as originally defined but are generally labour intensive and often require large amounts of starting genomic DNA. For example, HPLC measurement of total 5mC generally requires several micrograms of starting genomic DNA, whereas more contemporary ‘proxy’ approaches can be carried out using less than 100 ng. With the advent of highly specific antibodies to 5mC, more recent approaches such as ELISA are also available.

All other measures rely on the ascertainment of DNA methylation levels at a subset of genomic sites, with the underlying assumption that these reflect the global measure. The most popular involves the sampling of multiple copies of repetitive LINE (long interspersed numerical elements; mainly LINE-1) and SINE (short interspersed numerical elements; mainly Alu), amplified using degenerate primer sequences [6]. Together, these sites comprise upwards of 30% of human genomic DNA [7], but the actual number of potential sites of methylation within the genome remains lower. Less abundant satellite sequences have also been used to give insights to global methylation status, though these often represent even less of total genomic DNA by content [8]. Other methods use methylation sensitive (e.g. *HpaII*)/insensitive (*MspI*) restriction endonucleases [9]. Comparison of resulting genomic digestion patterns can yield insights regarding the level and distribution of 5mC within the genome, though this also represents a small fraction of total potential sites of DNA methylation. The luminometric methylation assay [10] or LUMA was adapted from these methods that measures fragments by a luminometric extension assay [11] with subsequent pyrosequencing.

An increasingly used proxy measure of overall methylation within a sample is often derived from genome-scale/wide methylation profiling. This is usually a mean or median methylation value of many thousands to millions of individual methylation values, spread throughout the genome and measured by beadarray, reduced representation, or whole genome bisulphite sequencing approaches.

## Considerations for interpretation

There are several important caveats to using any surrogate markers of global 5mC, primarily associated with the non-uniform nature of methylation within the human genome in association with genomic context. Firstly, when utilising a composite average (mean or median) measure derived from a large number of essentially unique sequences, it is important to note that most genomic DNA methylation is in fact found in repetitive elements scattered throughout the genome, such as transposons and endogenous retrovirus. Similarly, the *Hpa*II restriction site, often used to generate a proxy measure of global methylation, is enriched in high-density CpG islands comprising only ~12% of the total restriction sites in the human genome [12].

Secondly, repeat-based measures based on amplification using degenerate primer sequences generally only assess methylation at a subset of LINE or Alu elements, due to the range of sub-families of varying frequency and the large amount of sequence degeneration over time [13]. Although LINE-1 and Alu account for ~17 and 11% of the human genome [13], representing ~12 and 25% of all CpG dinucleotides respectively [14], only a subset of each can be interrogated by any given technique.

Finally, the mechanism of regulation of DNA methylation at different classes of unique and repetitive DNAs vary and therefore measuring one ‘type’ of methylation site is unlikely to be representative of global methylation levels. Direct comparison between approaches has been made, with varying results. In some in vitro cell line experiments, reduction in global methylation due to treatment by demethylating agents showed congruent results between Alu, LINE-1 and HPLC but not LUMA. In other cell lines, LINE-1, LUMA and HPLC yielded congruent levels of global hypomethylation. Generally, LINE-1 methylation status appears to correlate with HPLC measures more than Alu or LUMA, but not always, and this does not always correspond to a change in total global 5mC content [15]. The emerging picture is that no surrogate assay can accurately detect all biologically important differences in global 5mC content in all instances, with this needing to be ascertained on a case-by-case basis [16–19], particularly in the context of malignancy [20].

A recent comprehensive study explored whether global DNA methylation levels could be inferred from a combined measure of repeat-specific data [21]. Five alternative ‘global’ methylation approaches based on three technologies were employed including (i) high-performance liquid chromatography followed by mass spectrometry (HPLC-MS) [22] (ii) immunoquantification of global DNA methylation by ELISA [23] and, (iii) bisulfite pyrosequencing of a variety of different repetitive DNA elements (AluYb8/D4Z4/LINE/NBL2) [6, 24–26]. There was generally less agreement among the global DNA methylation assays across samples than with locus-specific DNA methylation assays, with the least reliable being the ELISA approach. A direct comparison of true global 5mC measures with average locus-specific (repeat-based) methylation showed a range of correlations, according to cell type and disease state. Interestingly, when combined with machine learning methods, repeat-specific assays reliably predicted sample-specific differences in true global 5mC levels [21].

## Biological relevance

It is important to note that several measures of ‘global’ methylation have been reported to vary in response to factors such as age, sex and cell composition. However, findings have been inconsistent. For example, total 5mC, as measured by HPLC in peripheral blood, has been found to be inversely associated with age [27, 28] whereas no association with age was found in analyses of LINE-1 [29, 30], Alu [30] or restriction-enzyme approaches [31], while another study using LUMA reported both gain and loss of methylation over time [32]. Further, other evidence suggests ageing-specific changes in Alu methylation in the absence of LINE-1 change. Such discrepancies indicate the possibility of heterogeneous changes of global methylation over time [33]. Indeed, changes in global methylation as measured by LUMA have identified age and tissue-specific effects in rats but not in the CG rich or promoter regions as previously utilised in other LUMA analyses [34]. In addition, age-specific changes are prominent near genes involved in metabolism indicating potential biological feasibility. Conversely, global methylation changes may not track with chronological age but as a result of functional decline [10], though the direction of causality is yet to be ascertained. A potential confounder of ageing-specific changes in global methylation has been linked to dietary composition including alteration in blood lipid profiles [35] and changes in nutritional content [36].

Distinguishing the type of measure employed is important in the case of disease association studies. For example, LINE-1 hypomethylation of peripheral blood, assessed prospectively, has been identified as a risk factor for a range of cancers [37], including bladder cancer [38]. Additionally, greater Alu methylation has been identified as a predictive biomarker for prostate cancer [39]. Increased CpG island DNA methylation in peripheral blood, as measured by LUMA, has been prospectively associated with an increased risk of breast cancer [40]. However, in the same analysis, LINE-1 methylation showed no association. Conversely, LINE-1 methylation varies in some prostate cancers in the absence of any measurable change in global 5mC content as assessed by HPLC [41].

Variations in the level of repeat-specific methylation are important, as ‘repeat-based’ hypomethylation has been implicated in the genomic instability associated with tumour progression and outcome [35, 42]. Further, repeat-based hypomethylation within a sample will likely have distinct functional consequences from hypomethylation assessed through largely gene-associated CpG sites, irrespective of the number of such sites assessed. In this way, the definition of global methylation shifts between ‘repeat-based’ methylation as an assessment of genomic stability to promoter-specific methylation, primarily implicated in gene regulation.

## Conclusions

It is important to clearly elaborate the context of DNA being used to measure methylation levels as different ‘types’ of DNA methylation are often uncoupled in terms of regulation. Most surrogate or proxy measures do not reflect global methylation (total 5mC content) as originally defined, and even though changes in repeat-specific measures may accurately reflect changes in global 5mC, this is likely to be system- and disease-specific.
